# BMC Plant Biology reviewer acknowledgement 2015

**DOI:** 10.1186/s12870-016-0733-4

**Published:** 2016-02-26

**Authors:** Julia Simundza

**Affiliations:** BioMed Central, 233 Spring Street, New York, NY 10013-1578 USA

## Abstract

The editors of BMC Plant Biology would like to thank all our reviewers who have contributed to the journal in Volume 15 (2015).

**Ahmed Abdel-Hadi**

Egypt

**Serena Aceto**

Italy

**Maricelis Acevedo**

USA

**Manuel Acosta**

Spain

**Lynn Adler**

USA

**Manu Agarwal**

India

**Parvaiz Ahmad**

India

**Firoz Ahmed**

India

**Riccardo Aiese Cigliano**

Spain

**Takashi Akagi**

Japan

**Salim Al-Babili**

Saudi Arabia

**Maria Albani**

Germany

**Victor Albert**

USA

**Alessandro Alboresi**

Italy

**Ruben Alcazar**

Spain

**Juan De Dios Alché**

Spain

**Ross Alexander**

UK

**Pablo Aleza**

Spain

**Md. Nasim Ali**

India

**Ricardo Alia**

Spain

**Gordon Allison**

UK

**Annapurna Devi Allu**

Germany

**Maria Maddalena Altamura**

Italy

**Charles Ampomah-Dwamena**

New Zealand

**Ajith Anand**

USA

**John Andralojc**

UK

**Fernando Andres Lalaguna**

Germany

**Pedro Aparicio-Tejo**

Spain

**Malli Aradhya**

USA

**Magdalena Arasimowicz-Jelonek**

Poland

**Patricio Arce-Johnson**

Chile

**Nagwa Aref**

Saudi Arabia

**Lirio Milenka Arevalo-Soliz**

USA

**A Argiriou**

Greece

**Jason Argyris**

Spain

**Hamed Arisha**

Egypt

**Grace Armijo**

Chile

**Rajeev Arora**

USA

**Stephanie Arrivault**

Germany

**Rosa Arroyo-García**

Spain

**Louise Arve**

Norway

**Sebastian Asurmendi**

Argentina

**Ross Atkinson**

New Zealand

**Belay Ayele**

Canada

**Brian Ayre**

USA

**João Lúcio Azevedo**

Brazil

**Kamran Azim**

Pakistan

**Nagesh Babu R**

India

**Andreas Bachmair**

Austria

**Agnieszka Bagniewska-Zadworna**

Poland

**Ranjit Prasad Bahadur**

India

**Samantha Baldwin**

New Zealand

**Daniel Ballhorn**

USA

**Matteo Ballottari**

Italy

**Dirk Balmer**

Switzerland

**Lois Banta**

USA

**Zhilong Bao**

USA

**Simon Barak**

Israel

**Abdelali Barakat**

USA

**Gregorio Barba-Espin**

Denmark

**Harbans Bariana**

Australia

**Matilde Barón Ayala**

Spain

**Amalia Barone**

Italy

**Rodolfo Barreiro**

Spain

**Blanca Estela Barrera**

Mexico

**Cristina Barrero Sicilia**

UK

**Craig Barrett**

USA

**Pedro Barros**

Portugal

**Cornelius Barry**

USA

**Dudy Bar-Zvi**

Israel

**Daniele Bassi**

Italy

**Nahla Bassil**

USA

**Giorgia Batelli**

Italy

**Jacqueline Batley**

Australia

**Eva Bauer**

Germany

**Stefan Bauer**

USA

**Emmanuelle Bayer**

France

**Perrin Beatty**

Canada

**Annette Becker**

Germany

**Diana Bellin**

Italy

**Catherine Bellini**

Sweden

**Abdelhafid Bendahmane**

France

**Noureddine Benkeblia**

Jamaica

**Dominique Bergmann**

USA

**Jamila Bernardi**

Italy

**Rex Bernardo**

USA

**Mark Bernards**

Canada

**Michael Bevan**

UK

**Rishikesh Bhalerao**

Sweden

**Yang Bian**

USA

**Melanie Bisson-Ritter**

Germany

**Oliver Bitz**

Germany

**Mark Bleackley**

Australia

**Eduardo Blumwald**

USA

**Natacha Bodenhausen**

Switzerland

**Christine Boettcher**

Australia

**María Del Carmen Bolarín Jiménez**

Spain

**Hanna Bolibok-Bragoszewska**

Poland

**Cordelia Bolle**

Germany

**Atle Bones**

Norway

**Ludovic Bonhomme**

France

**Dario Bonnetta**

Canada

**Philippa Borrill**

UK

**Julio Borsani**

Uruguay

**Jorunn Bos**

UK

**Maurice Bosch**

UK

**Paul Boss**

Australia

**Rebecca Boston**

USA

**Alessandro Botton**

Italy

**John Bowman**

Australia

**Cameron Bracken**

Australia

**Peter Bradbury**

USA

**James Bradeen**

USA

**Federico Brilli**

Italy

**Samuel Brockington**

UK

**Bettina Broeckling**

USA

**Patrick Brown**

USA

**Sigal Brown**

Israel

**Teerapong Buaboocha**

Thailand

**Hikmet Budak**

Turkey

**Pradeep Burma**

India

**Katharina Bürstenbinder**

Germany

**Mary Byrne**

Australia

**Caitlin Byrt**

Australia

**Yuanheng Cai**

USA

**Birsen Cakir**

Turkey

**Robin Cameron**

Canada

**Xiaofeng Cao**

China

**Alberto Carbonell**

Spain

**Maura Cardarelli**

Italy

**Francesca Cardinale**

Italy

**Anders Carlsson**

Sweden

**Fernando Carrari**

Argentina

**Bernard Carroll**

Australia

**Carla Caruso**

Italy

**Giorgio Casadoro**

Italy

**Jose Casaretto**

Chile

**Stuart Casson**

UK

**Simone Castellarin**

Canada

**Ariel Castro**

Uruguay

**Luigi Cattivelli**

Italy

**Valentina Cecchetti**

Italy

**David Chagne**

New Zealand

**Hao-Xun Chang**

USA

**Debasis Chattopadhyay**

India

**Rupal Chauhan**

India

**John Cheeseman**

USA

**Charles Chen**

USA

**Changbin Chen**

USA

**Wenrong Chen**

China

**Hong-Hwa Chen**

Taiwan

**Yaou Chen**

China

**Shilin Chen**

China

**Zhixiang Chen**

USA

**Zhiyuan Chen**

USA

**Ravindra Chibbar**

Canada

**Hyung-Taeg Cho**

Republic Of Korea

**Giltsu Choi**

South Korea

**Kang Chong**

China

**Surinder Chopra**

USA

**Pascal-Antoine Christin**

UK

**David Christopher**

USA

**Zhaoqing Chu**

China

**George Chuck**

USA

**Marta Cifuentes**

Spain

**Marion Clavel**

France

**Stephan Clemens**

Germany

**Jeremy Coate**

USA

**Patricia Coello**

Mexico

**Eva Collakova**

USA

**Nicholas Collins**

Australia

**Lucio Conti**

Italy

**Alex Costa**

Italy

**Fabrizio Costa**

Italy

**Laura Costantini**

Italy

**Pedro Crevillen**

Spain

**Andrew Charles Cuming**

UK

**Ann Cuypers**

Belgium

**Fatima Cvrckova**

Czech Republic

**Tomasz Czechowski**

UK

**Fabio M. Da Matta**

Brazil

**Olga Danilevskaya**

USA

**Yasuhiro Date**

Japan

**Swapan Datta**

India

**Kuntal De**

USA

**Paolo De Franceschi**

Italy

**Jose De Jesus Luna Ruiz**

Mexico

**Vincenzo De Luca**

Canada

**Ruud De Maagd**

Netherlands

**José De Vega Bartol**

UK

**Pasquale De Vita**

Italy

**Humberto Debat**

Argentina

**Thomas Defalco**

Canada

**Regine Delourme**

France

**Laurent Deluc**

USA

**Isabel Desgagné-Penix**

Canada

**Paul Devlin**

UK

**Christophe D'hulst**

France

**Gabriele Di Gaspero**

Italy

**Antonio Di Matteo**

Italy

**Maria Celeste Dias**

Spain

**Rebecca Dickstein**

USA

**Christine Dillmann**

France

**Antimo Dimaro**

Italy

**Yi Ding**

China

**Zj Ding**

China

**Gianfranco Diretto**

Italy

**Assaf Distelfeld**

Israel

**Christina Dixelius**

Sweden

**Richard Dixon**

USA

**Teresa Docimo**

Italy

**Agnès Doligez**

France

**Eva Dominguez**

Spain

**Claudio D'onofrio**

Italy

**Bryon Donohoe**

USA

**David Douches**

USA

**Georgia Drakakaki**

USA

**Ludovico Dreni**

China

**Patrick D'silva**

India

**Delin Duan**

China

**Alexandra Dubrovina**

Russian Federation

**Stefanie Dukowic-Schulze**

USA

**Marcello Duranti**

Japan

**Jonathan Duvick**

USA

**Beth Dyson**

UK

**Sue Ebeler**

USA

**Rudolf Eibach**

Germany

**Steven Eichten**

Australia

**Georgia Eizenga**

USA

**Mohamed El-Esawi**

Egypt

**Francesco Emanuelli**

Italy

**Takashi Endo**

Japan

**Eugenia Enfissi**

UK

**Maria Raffaella Ercolano**

Italy

**Carolina Escobar**

Spain

**Richard Espley**

New Zealand

**Raquel Esteban**

Spain

**Yoram Eyal**

Israel

**Hiroshi Ezura**

Japan

**Ahmed Faik**

USA

**Ahmad Fakhoury**

China

**Longjiang Fan**

China

**Congbing Fang**

China

**Eva Farre**

USA

**Alba Farre-Martinez**

UK

**Sara Farrona**

Ireland

**Attila Fehér**

Hungary

**Georg Felix**

Germany

**Xianzhong Feng**

China

**Felicidad Fernandez**

UK

**Paula Carmen Fernandez**

Argentina

**Angel Fernandez I Marti**

USA

**Alejandro Ferrando**

Spain

**Paulo Ferreira**

Brazil

**Albert Ferrer**

Spain

**Arthur Fett-Neto**

Brazil

**Ivo Feussner**

Germany

**Antonio Figueira**

Brazil

**Melania Figueroa**

USA

**Simone Fior**

Switzerland

**Andreas Fischer**

USA

**Henryk Flachowsky**

Germany

**Delphine Fleury**

Australia

**Eloise Foo**

Australia

**Brett Ford**

Australia

**Christopher Ford**

Australia

**Cinzia Formighieri**

USA

**Enrico Francia**

Italy

**Michael Francki**

Australia

**Wolfgang Frank**

Germany

**Keara Franklin**

UK

**Loreta Freitas**

Brazil

**Luciano Freschi**

Brazil

**Haya Friedman**

Israel

**Matthias Frisch**

Germany

**Yong-Fu Fu**

China

**Christian Fufezan**

Germany

**T Fukao**

USA

**Nobuko Fukino**

Japan

**Alexander Gabel**

Germany

**Agata Gadaleta**

Italy

**Jose Gadea**

Spain

**Selma Gago**

Germany

**Ab Gaikwad**

India

**Jean-Philippe Galaud**

France

**Gabor Galiba**

Hungary

**Daniel R Gallie**

USA

**Dongying Gao**

USA

**Caiji Gao**

Hong Kong

**Zhihong Gao**

China

**Liangliang Gao**

USA

**Nigel Gapper**

USA

**Dominique Garcia**

France

**Carlos García-Mata**

Argentina

**Melissa Gardner**

USA

**Amélie Gaudin**

USA

**Sonia Gazzarrini**

Canada

**Stanton Gelvin**

USA

**Jonathan Gent**

USA

**Emmanuel Geoffriau**

France

**Naomi Geshi**

Denmark

**Abelmon Da Silva Gesteira**

Brazil

**Fernando Geu Flores**

Denmark

**Michel Ghanem**

France

**Oula Ghannoum**

Australia

**Alberto Gianinetti**

Israel

**Susan Gibson**

USA

**Upinder Gill**

USA

**Matthew Gilliham**

Australia

**Philip Gilmartin**

UK

**Giovanni Giorio**

Italy

**Mike Giroux**

USA

**Thomas Givnish**

USA

**Donna Glassop**

Australia

**Cynthia Gleason**

Denmark

**María Luisa Gómez-Guillamón**

Spain

**Fernando Carlos Gómez-Merino**

Mexico

**Lei Gong**

China

**Zhenhui Gong**

China

**Daniel Gonzalez Mendoza**

Mexico

**M. Isabel González-Siso**

Spain

**William Gordon-Kamm**

USA

**Manje Gowda**

Kenya

**Thomas Gradziel**

USA

**Sean Graham**

Canada

**Lydia Gramzow**

Germany

**Heinrich Grausgruber**

Austria

**John Gray**

USA

**John Gray**

USA

**Christopher Grefen**

Germany

**Simon Groen**

USA

**Dennis Gross**

USA

**Ansgar Gruber**

Germany

**Kristina Gruden**

Slovenia

**Paul Gugger**

USA

**Sabine Guillaumie**

France

**Bilquees Gul**

Pakistan

**Patrick Gulick**

Canada

**Joel Gummer**

Australia

**Hui Guo**

USA

**Frank Guzman**

Brazil

**Flavia Guzzo**

Italy

**Bernd Hackauf**

Germany

**Michael Hahn**

USA

**Candace Haigler**

USA

**Pauliina Halimaa**

Finland

**Nathan Hall**

Australia

**Olivier Hamant**

France

**Mei Han**

Canada

**Fangpu Han**

China

**Dianwei Han**

USA

**Yuepeng Han**

China

**Kosuke Hanada**

Japan

**Robert Hancock**

UK

**Michael Handford**

Chile

**David Hannapel**

USA

**Paul Hasegawa**

USA

**George Haughn**

Canada

**Ludger Hausmann**

Germany

**Yuke He**

China

**Yuqing He**

China

**Guangyuan He**

China

**Hang He**

China

**Xin-Qiang He**

China

**Adrian Hegeman**

USA

**Martin Heil**

Germany

**Christopher Hendrickson**

USA

**Henk Hilhorst**

USA

**Andreas Hiltbrunne**

Germany

**Sally Hilton**

UK

**Heinz Himmelbauer**

Austria

**Dirk Hincha**

Germany

**Patricio Hinrichsen**

Chile

**Bertrand Hirel**

France

**Cory Hirsch**

USA

**Candice Hirsch**

USA

**Herman Hofte**

France

**Monica Hofte**

Belgium

**Saskia Hogenhout**

UK

**Gerrit Holighaus**

Germany

**Loreto Holuigue**

Chile

**Loren Honaas**

USA

**Zhi Hong**

China

**Tomoaki Horie**

Japan

**Walter Horst**

Germany

**Matthew Horton**

Austria

**David Horvath**

USA

**Yoichiro Hoshino**

Japan

**Xi-Lin Hou**

China

**Stephen H. Howell**

USA

**Li-Ching Hsieh**

Taiwan

**Yue-Ie Hsing**

Taiwan

**Songlin Hu**

USA

**Zhanming Hu**

China

**Jinping Hua**

China

**Zhihua Hua**

USA

**Hao-Jen Huang**

USA

**Baifei Huang**

China

**Yadong Huang**

USA

**Xi Huang**

China

**Matthew Hudson**

USA

**Gregorio Hueros**

Spain

**Irène Hummel**

France

**Steven Hussey**

South Africa

**Javier Ibáñez**

Spain

**Travis Idol**

USA

**Amy Iezzoni**

USA

**Kentaro Ifuku**

Japan

**Andrei Igamberdiev**

Canada

**Ernesto Igartua**

Estonia

**Yoko Iijima**

Japan

**Nikolaos Ioannidis**

Greece

**Sandra Irmisch**

Canada

**Atsushi Ishida**

Japan

**Maria Islas-Osuna**

Mexico

**Masaki Ito**

Japan

**Anjali Iyer-Pascuzzi**

USA

**Daniel J Sargent**

Italy

**Laura Jaakola**

Norway

**Guru Jagadeeswaran**

USA

**Mukesh Jain**

India

**Tiffany Jamann**

USA

**Sundaram Janarthanan**

India

**Robert Jansen**

USA

**Shelley Jansky**

USA

**Jose Jarillo**

Spain

**Wanquan Ji**

China

**Xian-Ling Ji**

China

**Xiaoyun Jia**

China

**Qidong Jia**

USA

**Yulin Jia**

USA

**Yuanqing Jiang**

China

**Wei Jiang**

China

**Yanping Jing**

China

**Jan Jirschitzka**

Germany

**Mark Jordan**

Canada

**Jesús V. Jorrín-Novo**

Spain

**Mary Prathiba Joseph**

Hungary

**Archana Joshi-Saha**

India

**Ricarda Jost**

Australia

**Ki-Hong Jung**

Republic Of Korea

**John Juvik**

USA

**Gudrun Kadereit**

Germany

**Shawn Kaeppler**

USA

**Sekhar Kambakam**

USA

**Mitsunobu Kamiya**

Japan

**Surya Kant**

Australia

**Michael Kantar**

USA

**Sanjay Kapoor**

India

**Beat Keller**

Switzerland

**Elizabeth Kellogg**

USA

**Marcia Kieliszewski**

USA

**Daniel Kierzkowski**

Germany

**Jeongwoon Kim**

USA

**Sun Yeou Kim**

South Korea

**Rod King**

Australia

**Tatjana Kleine**

Germany

**Christophe Klopp**

France

**Meyer Knut**

USA

**Michael Kobor**

Canada

**Gábor Kocsy**

Hungary

**Karl Heinz Kogel**

Germany

**Wouter Kohlen**

Netherlands

**Takao Komatsuda**

Japan

**Hyun Jo Koo**

USA

**Maarten Kooiker**

Netherlands

**Arkadiusz Kosmala**

Poland

**Jozef Kovacik**

Czech Republic

**Anja Krieger-Liszkay**

France

**Magdalena Krzymowska**

Poland

**Tiiu Kull**

Estonia

**Kundan Vijay Krishna Kumar**

India

**Sanjay Kumar**

India

**Santosh Kumar**

USA

**Sundaram Kuppu**

USA

**Miroslaw Kwasniewski**

Poland

**Carlos Labate**

Brazil

**John Labavitch**

USA

**Jinsheng Lai**

China

**Derek Thomas Anthony Lamport**

UK

**Jane Langdale**

UK

**John Larkin**

USA

**Charu Lata**

India

**David Lawlor**

UK

**Peter John Lea**

UK

**Esther Lechner**

France

**Jae-Hyeok Lee**

Canada

**Suk-Ha Lee**

South Korea

**Choong Hwan Lee**

South Korea

**Jong Kyu Lee**

South Korea

**David Legland**

France

**Johanna Leppälä**

Finland

**Gerhard Leubner-Metzger**

UK

**Jeffrey Leung**

France

**Christian Lexer**

Switzerland

**Qing Li**

USA

**Yongchun Li**

China

**Lin-Feng Li**

China

**Jinquan Li**

Germany

**Huihui Li**

China

**Maoteng Li**

China

**Ting Li**

USA

**Yongfang Li**

USA

**Wanlong Li**

USA

**Yongle Li**

Australia

**Yinxin Li**

China

**Zhengguo Li**

China

**Jiubo Liang**

China

**Yucai Liao**

China

**Diego Lijavetzky**

Argentina

**Jinxing Lin**

China

**Haijian Lin**

China

**Shanzhi Lin**

China

**Alexander Lipka**

USA

**Bao Liu**

China

**Shengyi Liu**

China

**Jianquan Liu**

China

**Hongtao Liu**

China

**Liangyu Liu**

China

**Gongshe Liu**

China

**Pei Liu**

China

**Renyi Liu**

China

**Shao-Lun Liu**

Taiwan

**Tongxian Liu**

China

**Zhongyuan Liu**

USA

**Maren Livaja**

Germany

**Danny Llewellyn**

Australia

**James Lloyd**

South Africa

**Alan Lloyd**

USA

**L Maria Lois**

Spain

**Ann Loraine**

USA

**Ping Lou**

USA

**Chung-An Lu**

Taiwan

**Nan Lu**

USA

**Jutta Ludwig-Müller**

Germany

**Lewis N. Lukens**

Canada

**John Lunn**

Germany

**Hongmei Luo**

China

**Feng Luo**

USA

**Alexander Lux**

Slovakia

**Martin Lysak**

Czech Republic

**Dinpow Ma**

USA

**Marco Maccaferri**

Italy

**Emma Mace**

Australia

**Eva Madrid Herrero**

Germany

**James Mahan**

USA

**Shamoni Maheshwari**

USA

**Yuko Makita**

Japan

**Giulia Malacarne**

Italy

**Pal Maliga**

USA

**Robert Malinowski**

Poland

**Mickael Malnoy**

Italy

**Giuseppe Mandolino**

Italy

**Alagu Manickavelu**

Japan

**Patricia Manosalva**

USA

**Yunxiang Mao**

China

**Felipe Maraschin**

Brazil

**Daniel Marino**

Spain

**David Marks**

USA

**José M Martinez-Zapater**

Spain

**Enrico Martinoia**

Switzerland

**Stefania Masci**

Italy

**Annaliese Mason**

Germany

**Esten Mason**

USA

**Patrick Masson**

USA

**Anna Maria Mastrangelo**

Italy

**Diane Mather**

Australia

**Marjori Matzke**

Taiwan Republic Of China

**Stephane Maury**

France

**Martin Mcainsh**

UK

**C. Robertson Mcclung**

USA

**Andrew Mccubbin**

USA

**Heather Mcfarlane**

Germany

**Rc Mcgarry**

USA

**Cecilia Mcgregor**

USA

**Cecilia Mcgregor**

USA

**Lee Meisel**

Chile

**David Mendoza**

USA

**Qingwei Meng**

China

**Zheng Meng**

China

**Tesfaye Mengiste**

USA

**Joachim Messing**

USA

**Pere Mestre**

France

**Ritesh Mewalal**

South Africa

**Louise Michaelson**

UK

**Diego Micheletti**

Italy

**Mehdi Mirzaei**

Australia

**Zbigniew Miszalski**

Poland

**Ortrun Mittelsten-Scheid**

Austria

**Karsten Mody**

Germany

**Wolfgang Moeder**

Canada

**Volker Mohler**

Germany

**Marta Molnar-Lang**

Hungary

**Paulo Monjardino**

Portugal

**Jonathan Monroe**

USA

**Pascal Montoro**

France

**Geoffrey Morris**

USA

**Panagiotis Moschou**

Sweden

**Claudio Moser**

Italy

**Gary Muehlbauer**

USA

**Kai Mueller**

Germany

**Siddique Muhammad-Aboobucker**

USA

**Karel Muller**

Czech Republic

**Rana Munns**

Australia

**Jorge Muschietti**

Argentina

**Alan Myers**

USA

**Sean Myles**

Canada

**Joshua Mylne**

Australia

**Kiyotaka Nagaki**

Japan

**Fares Najar**

USA

**Takahiro Nakamura**

Japan

**Kazuo Nakashima**

Japan

**Bharath Narayana**

USA

**Simona Nardozza**

New Zealand

**Annette Nassuth**

Canada

**Aura Navarro**

Germany

**Uwe Nehls**

Germany

**James Nelson**

USA

**Alexandre Nepomuceno**

Brazil

**Henry Nguyen**

USA

**Zhongfu Ni**

China

**Kare Lehmann Nielsen**

Denmark

**Tom Nielsen**

Denmark

**Jennifer Normanly**

USA

**Michitaka Notaguchi**

Japan

**Dietrich Ober**

Germany

**Mary O'connell**

USA

**Thomas Odong**

Uganda

**Yoshiyuki Ogata**

Japan

**Kyoko Ohashi-Ito**

Japan

**John Ohlrogge**

USA

**Akira Oikawa**

Japan

**Sezer Okay**

Turkey

**Richard Olmstead**

USA

**Youko Oono**

Japan

**Etti Or**

Israel

**Jamie O'rourke**

USA

**Doug Orr**

UK

**Thomas Ott**

Germany

**Carl-Otto Ottosen**

Denmark

**Francois Ouellet**

Canada

**Yoshihiro Ozeki**

Japan

**Hyun-Sook Pai**

Republic Of Korea

**Junsong Pan**

China

**Girdhar Pandey**

India

**Ivan Paponov**

Norway

**Swarup Parida**

India

**Sunghun Park**

USA

**Wonkeun Park**

USA

**Claudio Pastenes**

Chile

**Victoria Pastor**

Switzerland

**Vikas Patade**

India

**John Patrick**

Australia

**Mario Enrico Pè**

Italy

**Ales Pecinka**

Germany

**Christie Peebles**

USA

**Jason Peiffer**

USA

**Tibor Penchan**

USA

**Dingxiang Peng**

China

**Shiqing Peng**

China

**Michele Perazzolli**

Italy

**Luiz Filipe Pereira**

Brazil

**Miguel Perez-Amador**

Spain

**Rajesh Perianayagam**

USA

**Staffan Persson**

Australia

**Elena Pestsova**

Germany

**Maike Petersen**

Germany

**Daniel Peterson**

USA

**Mario Pezzotti**

Italy

**Td Pham**

Sweden

**Corné Pieterse**

Netherlands

**Ken Piller**

USA

**Christoph Plieth**

Germany

**Kim Plummer**

Australia

**Scott Poethig**

USA

**Filip Pošćić**

Italy

**Carlo Pozzi**

Italy

**Kalika Prasad**

India

**Estelle Proux-Wéra**

Sweden

**Jo Putterill**

New Zealand

**Weiqiang Qian**

China

**Sheng Qiang**

China

**Song Qin**

China

**Yuan Qin**

China

**Wang Qing-Feng**

China

**Marian Quain**

Ghana

**Marie-Christine Quillet**

France

**Habibur Rahman**

Canada

**Tenhaken Raimund**

Austria

**Om Rajora**

Canada

**Harsh Raman**

Australia

**Christopher Randle**

USA

**Guodong Rao**

China

**Susanne Rasmussen**

New Zealand

**Pascal Ratet**

France

**Mark Rausher**

USA

**Kundapura Ravishankar**

India

**Umesh Reddy**

USA

**Margaret Redinbaugh**

USA

**Kent Redman**

USA

**Tony Reglinski**

New Zealand

**James Reid**

Australia

**Jochen Reif**

Germany

**Simon Renny-Byfield**

USA

**Stefan Rensing**

Germany

**Ralf Reski**

Germany

**Davina Rhodes**

USA

**Glen Ritchie**

USA

**Renata Rivera-Madrid**

Mexico

**Alison Roberts**

USA

**Ms Roder**

Germany

**Pedro Rodriguez**

Spain

**Hilary Rogers**

UK

**Hannetz Roschzttardt**

USA

**Daryl Rowan**

New Zealand

**Stuart Roy**

Australia

**Aryadeep Roy Choudhury**

India

**Joaquín Royo**

Spain

**Yong-Ling Ruan**

Australia

**Brigitte Ruge-Wehling**

Germany

**Markus Ruhsam**

UK

**Scott Russell**

USA

**Kamil Ruzicka**

Czech Republic

**Hojjatollah Saeidi**

Islamic Republic Of Iran

**Lingaraj Sahoo**

India

**Yusuke Saijo**

Japan

**Cyrille Saintenac**

France

**Yasuhito Sakuraba**

Republic Of Korea

**Silvio Salvi**

Italy

**Jozef Samaj**

Czech Republic

**Eduardo Sanchez-Bermejo**

UK

**Juan Luis Santos Coloma**

Spain

**Luis Santos Del Blanco**

Switzerland

**Gautam Sarath**

USA

**Daniel Sargent**

Italy

**Tomasz Sarnowski**

Poland

**Nobuhiro Sasaki**

Japan

**Hidenori Sassa**

Japan

**Masa Sato**

Japan

**Sampurna Sattar**

USA

**Margret Sauter**

Germany

**Christopher Sauvage**

France

**Andreas Savvides**

Cyprus

**Samir Sawant**

India

**Mary Schaeffer**

USA

**Robert Schaffer**

New Zealand

**Ulrich Schaffrath**

Germany

**Gert Schansker**

Hungary

**Klaus-Dieter Scharf**

Germany

**Henk Schat**

USA

**Andreas Schiermeyer**

Germany

**Jos Schippers**

Germany

**Axel Schmidt**

Germany

**Romy Schmidt**

Germany

**Leigh Schmidtke**

Australia

**Bob Schmitz**

USA

**Korbinian Schneeberger**

Germany

**Thorsten Schnurbusch**

Germany

**Chris-Carolin Schoen**

Germany

**Daniel Scholes**

USA

**Henk Schouten**

Netherlands

**Kathrin Schrick**

USA

**Julian Schroeder**

USA

**Ingo Schubert**

Germany

**Meredith Schuman**

Germany

**Markus Schwarzländer**

Germany

**Barry Scott**

New Zealand

**Paul Scott**

USA

**David Secco**

Australia

**Armand Seguin**

Canada

**Verena Seidl-Seiboth**

Austria

**Georg Seifert**

Austria

**Agnieszka Sendek**

Germany

**Ramón Serrano**

Spain

**Sergey Shabala**

Australia

**Dilip Shah**

USA

**Libo Shan**

USA

**Timothy F. Sharbel**

Germany

**Pradeep Sharma**

India

**Sherif Sherif**

Canada

**Huazhong Shi**

USA

**Kai Shi**

China

**Hai-Yan Shi**

China

**Takashi Shiina**

Japan

**Toshiharu Shikanai**

Japan

**Behrouz Shiran**

Islamic Republic Of Iran

**Shin-Han Shiu**

USA

**Huixia Shou**

China

**Rakesh Shukla**

India

**Cristina Silvar**

Spain

**Joaquim Silveira**

Brazil

**Anil Kumar Singh**

India

**Bramad Singh**

India

**Sukhwinder Singh**

Mexico

**Alok Sinha**

India

**Daniel Skinner**

USA

**Gancho Slavov**

UK

**R. Keith Slotkin**

USA

**Alison Smith**

UK

**David Smyth**

Australia

**Moon-Soo Soh**

South Korea

**Juan Solomon**

USA

**Pamela Soltis**

USA

**Prakit Somta**

Thailand

**Jingyuan Song**

China

**Qijian Song**

USA

**Jie Song**

China

**Kai Song**

China

**Shuhui Song**

China

**Songquan Song**

China

**Wim Soppe**

Germany

**Braulio Soto-Cerda**

Chile

**Pawe Sowinski**

Poland

**Erin Sparks**

USA

**Raul Sperotto**

Brazil

**Cornelia Spetea-Wiklund**

Sweden

**Wolfgang Spielmeyer**

Australia

**Charles Spillane**

Ireland

**Jennifer Spindel**

USA

**Gary Stacey**

USA

**Yannick Städler**

Austria

**Joshua Stein**

USA

**David B. Stern**

USA

**Benjamin Stich**

Germany

**Helena Storchova**

Czech Republic

**Jake Stout**

Canada

**Nathaniel Street**

Sweden

**Robert Stupar**

USA

**Zhen Su**

China

**Xiaohua Su**

China

**Subhashree Subramanyam**

USA

**Mi Chung Suh**

Republic Of Korea

**Genlou Sun**

Canada

**Wenxian Sun**

China

**Mengxiang Sun**

China

**R.M. Sundaram**

India

**Sridevi Sureshkumar**

Australia

**Robert Swanson**

USA

**Lee Sweetlove**

UK

**Crystal Sweetman**

Australia

**Vaughan Symonds**

New Zealand

**Laszlo Szabados**

Hungary

**Francisco Tadeo**

Spain

**Teruaki Taji**

Japan

**Tomas Takac**

Czech Republic

**Hideyuki Takahashi**

Japan

**Seiji Takahashi**

Japan

**Shigeo Takumi**

Japan

**Cecilia Tamborindeguy**

USA

**Kenichi Tamura**

Japan

**Bao-Cai Tan**

China

**Masaru Tanaka**

Japan

**Tsuyoshi Tanaka**

Japan

**Weihua Tang**

China

**Jihua Tang**

China

**Yuhong Tang**

USA

**Sithichoke Tangphatsornruang**

Thailand

**Georgia Tanou**

Greece

**Dayun Tao**

China

**Evangelos Tatsis**

UK

**Nicolas Taylor**

Australia

**Sheng Teng**

China

**Sachin Teotia**

USA

**Ian Tetlow**

Canada

**Nguyen Phuong Thao**

Viet Nam

**Flavia Thiebaut**

Brazil

**Stephen Thomas**

UK

**Richard Thompson**

France

**Gregory Thyssen**

USA

**Zhixi Tian**

China

**Denise Tieman**

USA

**Peter Tiffin**

USA

**Marja Timmermans**

USA

**Alain Tissier**

Germany

**Takayuki Tohge**

Germany

**Hüseyin Tombuloğlu**

Turkey

**Alessandro Tondelli**

Italy

**Chaobo Tong**

China

**Rubén Torices**

Portugal

**Livio Trainotti**

Italy

**Maurizio Trovato**

Italy

**Veronica Truniger**

Spain

**Aleksandra Trzewik**

Poland

**Hirokazu Tsukaya**

Japan

**Chih-Wei Tung**

Taiwan

**Franziska Turck**

Germany

**Ismail Turkan**

Turkey

**Rickie Turley**

USA

**Monique Turmel**

Canada

**G Turner**

USA

**Daniel Tyteca**

Belgium

**Cristobal Uauy**

UK

**M Udayakumar**

India

**Rita Ulloa**

Argentina

**Kristian Ullrich**

Germany

**Santosh Upadhyay**

India

**Jyothilakshmi Vadassery**

India

**Giampiero Valè**

Italy

**Estela Valle**

Argentina

**Claudio Valverde**

Argentina

**Clemens Van De Wiel**

Netherlands

**Harrold Van Den Burg**

Netherlands

**Renier Van Der Hoorn**

Germany

**Marna Van Der Merwe**

Australia

**Joost Van Heerwaarden**

Netherlands

**Marie-Anne Van Sluys**

Brazil

**Klaas Vandepoele**

Belgium

**João Carlos Serafim Varela**

Portugal

**Lakshminarayana Vemireddy**

India

**Vivek Verma**

UK

**Paul Verslues**

USA

**Paul Verslues**

Taiwan

**Alessandro Vezzi**

Italy

**Filipe Victoria**

Brazil

**Vikram Virdi**

Belgium

**Paola Vittorioso**

Italy

**Giannina Vizzotto**

Italy

**Konstantinos Vlachonasios**

Greece

**Boris Voigt**

Germany

**Albrecht Von Arnim**

USA

**Maria Von Korff**

Germany

**Antje Von Schaewen**

Germany

**Eric Von Wettberg**

USA

**Eva Vranova**

Slovakia

**Wim Vriezen**

Netherlands

**Jason Wallace**

USA

**Christopher Wallis**

USA

**Michael H. Walter**

Germany

**Xiuling Wang**

China

**Yu Fu Wang**

China

**Meinan Wang**

USA

**Richard Wang**

USA

**Lei Wang**

China

**Xiue Wang**

China

**Xiu-Jie Wang**

China

**Yonghong Wang**

China

**Yi Wang**

China

**Marilyn Warburton**

USA

**John Ward**

USA

**Claus Wasternack**

Germany

**Mutsumi Watanabe**

Germany

**Fabrice Wattebled**

France

**Melanie Wecker**

Australia

**Donald Weeks**

USA

**Claus Weinholdt**

Germany

**Jim Weller**

Australia

**Ralf Welsch**

Germany

**Neele Wendler**

Germany

**Yiqun Weng**

USA

**Diego Wengier**

USA

**Elizabeth Weretilnyk**

Canada

**Randall Weselake**

Canada

**David Willmot**

USA

**Joe Win**

UK

**Astrid Wingler**

Ireland

**Darren Wong**

Canada

**Sek-Man Wong**

Singapore

**Margeret Woodhouse**

USA

**Zhiqiang Wu**

USA

**Jian Wu**

China

**Xiangyuan Wu**

China

**Tao Xia**

China

**Xinli Xia**

China

**Yun Xiang**

China

**Qingqing Xie**

China

**Haiping Xin**

China

**Yongzhong Xing**

China

**Han Xing**

China

**Tou Cheu Xiong**

France

**Yong Xu**

China

**Zhao-Shi Xu**

China

**Zhengyi Xu**

China

**Gang-Ping Xue**

Australia

**Liangjiao Xue**

USA

**Gitanjali Yadav**

India

**Toshihiko Yamada**

Japan

**J Yan**

China

**Yueming Yan**

China

**Yajun Yang**

China

**Xiyan Yang**

China

**Changqing Yang**

China

**Ming Yang**

USA

**Qing-Chuan Yang**

China

**Ling Yang**

China

**Yongping Yang**

China

**Zhi Min Yang**

China

**Ziyin Yang**

China

**Qing Ye**

China

**Zhuoliang Ye**

USA

**Yanhai Yin**

USA

**Tiejin Ying**

China

**John Yoder**

USA

**Jun Yu**

China

**Zheng Yuan**

China

**Lorenzo Zacarias**

Sri Lanka

**Latiffah Zakaria**

Malaysia

**Anita Zamboni**

Italy

**Jurgen Zeier**

Germany

**Dali Zeng**

China

**Philipp Zerbe**

USA

**Poliane Zerbini**

Brazil

**Bosen Zhang**

USA

**Tianzhen Zhang**

China

**Dadong Zhang**

USA

**Jin-Zhi Zhang**

China

**Lingyun Zhang**

China

**Junli Zhang**

USA

**Junli Zhang**

USA

**Wenjun Zhang**

USA

**Xunzhong Zhang**

USA

**Huijuan Zhang**

China

**Hukun Zhang**

China

**Kewei Zhang**

China

**Lei Zhang**

China

**Zhang Zhang**

China

**Jiao-Lin Zhang**

China

**X Zhang**

USA

**Chengsong Zhao**

USA

**Yunde Zhao**

USA

**Yunjun Zhao**

USA

**Meixia Zhao**

USA

**Mingui Zhao**

China

**Bo Zheng**

China

**Binglian Zheng**

China

**Wenguang Zheng**

USA

**Jun Zheng**

China

**Yun Zheng**

China

**Dao-Xiu Zhou**

China

**Man Zhou**

USA

**Wangsheng Zhu**

Germany

**Jing Zhuang**

China

**Khurram Ziaf**

Pakistan

**Elizabeth Zimmer**

USA

**Matthew Zinkgraf**

USA

**Piotr Ziolkowski**

Poland

**Cheng Zou**

China

**Rita Zrenner**

Germany

**Agustin Zsogon**

Brazil

**Jianru Zuo**

China

**Kaijing Zuo**

China

